# The Potential Protective Effect of Iridoid Glycosides Isolated From *Osmanthus fragrans* Seeds Against the Development of Immune Liver Injury in Mice

**DOI:** 10.3389/fphar.2021.760338

**Published:** 2021-11-08

**Authors:** Yuchen Zhang, Feng Xiao, Qiqi Zhou, Tingting Diao, Meng Zhang, Dongyang Liu, Zhuowen Wang, Ting Huang, Yupei Wu, Yuting Bai, Qing Min

**Affiliations:** ^1^ School of Pharmcy, Hubei University of Science and Technology, Xianning, China; ^2^ School of Biological and Pharmaceutical Engineering, Xinyang Agricultural and Forestry University, Xinyang, China

**Keywords:** *Osmanthus fragrans*, iridoid glycosides, content determination, HPLC, liver injury

## Abstract

**Objective:** The iridoid glycosides were extracted and separated from *Osmanthus fragrans* seeds, and the potential protective effect of *Osmanthus fragrans* seed extract on concanavalin A-induced immune liver injury in mice was studied.

**Methods:** *Osmanthus fragrans* seeds were extracted by 95% ethanol reflux. Then, the iridoid glycosides were enriched by extraction refined through petroleum ether (60°C–90°C), ethyl acetate, and water-saturated n-butanol in sequence, so as to purify the n-butanol part (*Osmanthus fragrans* seed’s n-butanol extraction, OFSN) by macroporous resin. Specnuezhenide and Nuezhenoside G13 were used as the reference substances to determine the concentration of iridoid glycosides by HPLC. On this basis, a mouse immune liver injury model was established by tail intravenous concanavalin A (20 mg/kg); the contents of serum ALT, AST, IFN-γ, and TNF-α and the contents of liver tissue MDA and SOD were determined; the pathological changes of the liver by HE staining were observed; and the expression levels of p38MAPK and p-p38mapk in liver tissue were detected by WB.

**Results:** The linearity, precision, repeatability, recovery, and stability of HPLC all met the requirements by validating with the methodology. The contents of Specnuezhenide and Nuezhenoside G13 in the n-butanol extracts were 39.20% and 39.88%, respectively. Actually, their contents can reach up to 82.56% and 87.9% after being purified by macroporous resin. The results of animal experiments show that OFSN could significantly reduce the liver and spleen index, reduce the ALT and AST contents in plasma and the MDA content in liver tissue, and then increase the SOD content. Besides, OFSN could also reduce the plasma IFN-γ and TNF-α levels. The HE staining result indicates that the pathological changes in the liver tissues of mice treated with OFSN are alleviated to different degrees while the WB result suggests that OFSN could significantly inhibit the expression of p-p38mapk.

**Conclusion:** *Osmanthus fragrans* seeds are rich in iridoid glycosides, which has a good protective effect on mouse immune liver injury caused by concanavalin A. The mechanism may be related to inhibiting the phosphorylation of p38MAPK, inhibiting the release of inflammatory mediators, improving the antioxidant capacity of liver cells, and weakening the occurrence of lipid peroxidation.

## Introduction

*Osmanthus fragrans* Lour. are evergreen shrubs or small trees of the Oleaceae family, which blossom in the autumn and bear fruit in the spring of the following year. At present, people mainly develop the value of their flowers in the field of food, medicine, and cosmetics but miss the seeds. The result is a large number of *Osmanthus fragrans* seeds scattered on the ground, causing not only a certain amount of pollution to the environment but also a waste of resources. However, *Osmanthus fragrans* seeds are a traditional Chinese medicine, beneficial to liver, stomach, and other diseases. According to the “Compendium of Materia Medica” and “Jiangsu Materia Medica,” *Osmanthus fragrans* seeds have liver- and stomach-protective effects (Nanjing Pharmaceutical College, 1965; Li, 2007). Modern research shows that *Osmanthus fragrans* seeds contain a lot of active chemical components (Wang et al., 2006; Pan et al., 2009; Yin et al., 2013; Liao et al., 2018; Liao et al., 2019), such as iridoid glycosides, salidroside, oleanolic acid, ursolic acid, flavonoids, and volatile oil. Among them, iridoid glycosides are the landmark components of Oleaceae plants (Jensen et al., 2002), which have a variety of biological activities such as anti-inflammatory and analgesic, hepatoprotective and cholagogic, bacteriostatic, sedative, antihypertensive, and antitumor (Huang et al., 2011; Jawaid et al., 2015; Ouyang et al., 2015; Tang et al., 2015). Additionally, there are about 10 kinds of iridoid glycosides that have been isolated from *Osmanthus fragrans* seeds, including nuezhenoside, specnuezhenide, isonuezhenide, nuezhenoside G13, oleoside, oleoside dimethyl ester, and strychnine glycosides, among which the contents of specnuezhenide, isonuezhenide, and nuezhenoside G13 are higher (Yang et al., 2013).

Immune liver injury is a chronic progressive liver inflammatory disease, which is mediated by an autoimmune response, accompanied by clinical symptoms such as elevated serum aminotransferase, high γ-globulinemia, and positive autoantibody, which can rapidly develop into liver cirrhosis and liver failure in severe cases (Zhang et al., 2018). Currently, the clinical treatment involves using glucocorticoid alone or combining it with azathioprine. However, the long-term use of hormone drugs has adverse effects on the body, such as osteoporosis, thrombotic stroke or MI, and infection (van der Goes et al., 2016; Wilson et al., 2019). Thus, it is important to find safer and more effective new drugs. According to the research in recent years, traditional Chinese medicine has exhibited certain activities in the treatment of liver injury diseases, especially iridoid glycosides (Tan et al., 2017).

In this study, iridoid glycosides were first extracted from *Osmanthus fragrans* seeds and the content was determined by HPLC. Then, the mouse immune liver injury model was established by tail intravenous concanavalin A to study the protective effect of iridoid glycosides on immune liver injury in mouse liver. This work will not only promote the in-depth study of treatment of liver injury but also have a great significance to the development and utilization of *Osmanthus fragrans* resources in Xianning city.

## Materials and methods

### *Osmanthus fragrans* seeds

*Osmanthus fragrans* fruits were collected from the campus of Hubei University of Science and Technology (Xianning, Hubei, China) in April to May and were identified by Dr. Qing Min. The plant specimens were no. 20160420-20160513, according to a YY/MM/DD system used in our lab. The pericarp and flesh were removed, and the *Osmanthus fragrans* seeds were cleaned and dried at 60°C. Then, the seeds were crushed and sifted through a 100-mesh sieve.

### Extraction of iridoid glycosides from *Osmanthus fragrans* seeds

A total of 1,410 g crushed dried *Osmanthus fragrans* seeds were taken and soaked with petroleum ether (60°C–90°C, the ratio of material to liquid is 1:10) (Oubokai, Tianjin, China) for 48 h, during which the *Osmanthus fragrans* seeds were in full contact with petroleum ether (60°C–90°C) by moderate shaking every 12 h. We separated the filtrate and the residue, concentrated the filtrate to extract the petroleum ether, and dried the residue to get rid of petroleum ether. Then, we performed reflux extraction by 95% ethanol (Oubokai, Tianjin, China) (the ratio of material to liquid is 1:20) for three times (2 h each time), combined the extracting solution, and extracted the *Osmanthus fragrans* seeds’ ethanol (OFSE, the yield of 759.80 g) by vacuum concentration (DZF-6050 vacuum drying oven, Shanghai Boxun, Shanghai, China). Afterward, OFSE was suspended by adding an appropriate amount of water and extracted it with petroleum ether (60°C–90°C), ethyl acetate (Oubokai, Tianjin, China), and water-saturated n-butanol (Sinopharm, China) (each for three times). The extracting solution was collected, combined, and concentrated to obtain condensed extraction.

*Osmanthus fragrans* seed’s n-butanol extraction (OFSN, the yield of 200.0 g) was purified by D101 macroporous resin (Boshi, Shanghai, China) and eluted with H_2_O, 10% ethanol, 30% ethanol, 50% ethanol, 70% ethanol, and 95% ethanol in sequence. Then, each eluent was collected and concentrated to obtain each extraction.

### Determination of iridoid glycosides from *Osmanthus fragrans* seeds by HPLC

Specnuezhenide and Nuezhenoside G13 (Yilin, Shanghai, China) were used as the reference substances to determine the concentration of iridoid glycosides by HPLC (Agilent 1220, Agilent Technology Co. Ltd., Santa Clara, CA, USA).

#### Preparation of solution

**Preparation of reference solution:** 10 mg of Specnuezhenide and Nuezhenoside G13 was weighed (BP211D, Sartorius, Göttingen\, Germany), respectively, and added in 1,000 μl chromatographic methanol (HY Biocare Chem, Chiayi City, Taiwan) to dissolve. Then, we shook it well. The reference reserve solutions of Specnuezhenide and Nuezhenoside G13 were prepared at a concentration of 10.00 mg/ml.

**Preparation of mixed reference solution:** Precisely pipetted 100 μl of reference reserve solution of Specnuezhenide and Nuezhenoside G13 into the same 10-ml volumetric flask, diluted with chromatographic methanol to volume, and mixed.

**Preparation of sample solution:** 10 mg of *Osmanthus fragrans* seed extraction [*Osmanthus fragrans* seeds’ ethanol extraction (OFSE), *Osmanthus fragrans* seed’s n-butanol extraction (OFSN), 30% ethanol elution portion (OFSN-3), 50% ethanol elution portion (OFSN-4)] was extracted into the 10-ml volumetric flask. Then, it was dissolved and diluted with chromatographic methanol to volume and mixed then filtered with a 0.45-μm microporous membrane.

#### Determination of iridoid glycosides by HPLC

Sample solution, Specnuezhenide and Nuezhenoside G13 reference solution, and methanol were used as the solvent. UV scanning (Gary 50 cone, Varian, Palo Alto, CA, USA) was performed at 200–400 nm to obtain the maximum absorption wavelength, which was used as the detection wave for HPLC. Then, the content of iridoid glycosides from *Osmanthus fragrans* seeds was determined by an external standard method.

**FIGURE 1:**
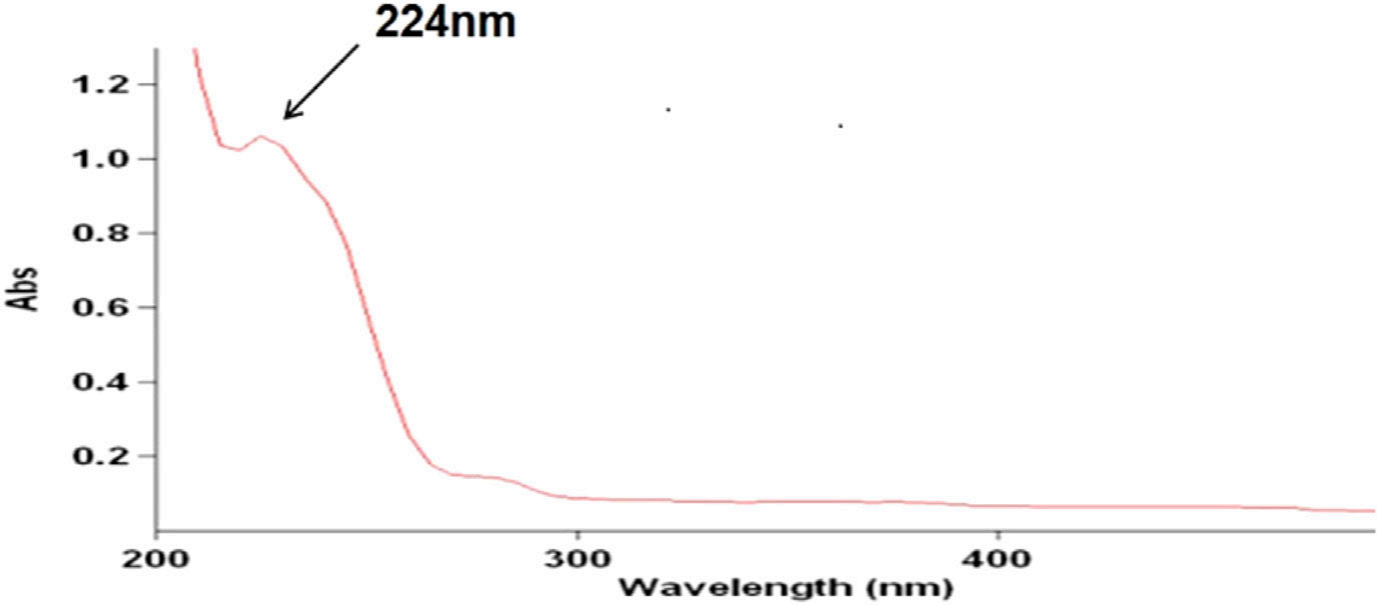
The ultraviolet absorption spectrum of *Osmanthus fragrans* seeds extraction.

The chromatographic conditions are as follows: chromatographic column, Eclipse XDB-C18 (4.6 mm × 250 mm, 5 μm) (Agilent Technology Co., Ltd.); mobile phase, water(A)-methanol(B); gradient elution, 0–10 min, 70% A→60% A; 10–35 min, 60% A→40% A; flow rate, 1 ml/min; column temperature, 30°C; detection wavelength, 224 nm (Figure 1: the ultraviolet absorption spectrum of *Osmanthus fragrans* seed extraction); amount of injection, 5 μl.

#### HPLC methodology validation

**System adaptability test:** 5 μl of the mixed reference solution was precisely pipetted, the retention time of Specnuezhenide and Nuezhenoside G13 was recorded, and the chromatographic peak shapes were observed according to the chromatographic conditions under item *Determination of Iridoid Glycosides by HPLC*.

**Linearity and range:** 25, 50, 100, 200, 300, 400, and 500 μl of Specnuezhenide and Nuezhenoside G13 reference reserve solution were precisely pipetted, into the 10-ml volumetric flask. Then, they were diluted with chromatographic methanol to volume and mixed. Five microliters of the above test solution was precisely pipetted for testing according to the chromatographic conditions under item *Determination of Iridoid Glycosides by HPLC*, and the peak area was recorded. Next, the standard curve was drawn with the reference concentration as the abscissa and the peak area as the ordinate, and the linear relationship between the sample concentration and the peak area was calculated.

**Precision test:** 5 μl of the mixed reference solution of Specnuezhenide and Nuezhenoside G13 was precisely pipetted and injected six times continuously. The chromatogram was recorded, and the relative standard deviation (RSD) of the peak area was calculated. An RSD less than 2% indicates good precision.

**Repeatability test:** 10 mg of OFSN for six parts was precisely weighed, and the test solution was prepared according to item *Preparation of Solution* and determined according to the chromatographic conditions under item *Determination of Iridoid Glycosides by HPLC*. Then, the Specnuezhenide and Nuezhenoside G13 content was calculated by the external standard method, as well as the RSD of the content. An RSD less than 2% indicates good repeatability.

**Recovery test:** 10 mg of OFSN was precisely weighed, and a moderate amount of the mixed reference solution of Specnuezhenide and Nuezhenoside G13 was pipetted to prepare three concentration gradients of low (80%), medium (100%), and high (120%) concentrations. Five microliters of the above test solution was precisely pipetted for testing according to the chromatographic conditions under item *Determination of Iridoid Glycosides by HPLC*. Then, the recovery rate and RSD of Specnuezhenide and Nuezhenoside G13 were calculated. An RSD less than 2% indicates good recovery.

**Stability test:** OFSN was weighed to prepare the sample solution according to item *Preparation of Solution*, the sample solution was placed at room temperature, and 5 μl of the sample solution was sampled at 0, 8, 16, and 36 h, for testing according to the chromatographic conditions under item *Determination of Iridoid Glycosides by HPLC*. Then, the Specnuezhenide and Nuezhenoside G13 content was calculated by the external standard method, as well as the RSD. An RSD less than 2% indicates good stability.

### Experimental animals

Sixty male Kunming mice (18–22 g) were provided by Liaoning Changsheng Biotechnology Co., Ltd., license number: SCXK (Liao) 20150001. All experimental protocols and procedures were conducted in accordance with the Guide for the Care and Use of Laboratory Animals published by the National Institutes of Health (NIH publication 85-23, revised 1996) and approved by the Committee of Experimental Animals of Hubei University of Science and Technology. The experimental animals were raised in the animal room of the School of Pharmacy, Hubei University of Science and Technology, with a cleaning grade of 20°C and a humidity of 55%. Food and water were available *ad libitum*.

### Preparation of immune liver injury model

Sixty male Kunming mice were randomly divided into six groups (*n* = 10), the control group, the immune liver injury model group, the positive control group (prednisolone acetate 6.4 mg/kg), and the OFSN low (125 mg/kg), OFSN medium (250 mg/kg), OFSN high (500 mg/kg) dose groups. The animals were fed with routine chow diet throughout (Wanqianjiaxing, Wuhan, China). All mice were given drugs by intragastric administration once a day for 10 days, and the control and model groups were treated with an equal volume of saline. After the last intragastric administration for 1 h, the control group was given normal saline (10 ml/kg) by tail vein injection, and the other groups were all given concanavalin A (20 mg/kg, dissolved with normal saline) to establish the immune liver injury model (Zhang et al., 2013). Then, all mice were fasted and drank freely.

### Taken specimens

Blood was collected from the eyeball after modeling for 12 h, then the mice were executed by cervical dislocation and the liver and spleen were placed on the ice. The liver and spleen were rinsed in 4°C normal saline to remove blood stains, dried with filter paper, and weighed, and the liver and spleen indexes were calculated. Then, the whole blood was centrifuged (LaboGene, Lillerød, Denmark) at 3,000 r/min for 10 min, the supernatant was carefully absorbed, and the plasma was kept for the following assay.

### ALT, AST, TNF-α, and IFN-γ assay of the plasma

The glutamic pyruvic transaminase (ALT, GPT), glutamic oxalacetic transaminase (AST, GOT) (Nanjing Jiancheng, Nanjing, China), tumor necrosis factor-α (TNF-α), and interferon-γ (IFN-γ) (Lianke, Hangzhou, China) of the plasma were measured using the associated detection kits according to the manufacturer’s instructions in different reagent kits.

### MDA and SOD assay of the liver tissues

The liver tissues were rinsed in 4°C normal saline to remove blood stains and dried with filter paper. About 0.1 g of liver tissue was weighed and homogenized (1:9, w/v) in normal saline with an electric homogenizer. Then, the homogenate was centrifuged at 3,000 r/min for 15 min to collect the supernatant. The superoxide dismutase (SOD) and malondialdehyde (MDA) (Nanjing Jiancheng, Nanjing, China) were measured using the associated detection kits according to the manufacturer’s instructions in different reagent kits.

### Histological analysis of the liver

The sample of liver tissue blocks (about 0.2 g) was taken and fixed with 4% buffered paraformaldehyde for 24 h. The tissue blocks were rinsed with running water. After dehydration and transparency (Yaguang, Hubei, China), the tissue blocks were embedded in paraffin (Yaguang, Hubei, China) and sectioned into 4-µm thickness (RM2235, Leica, Wetzlar, Germany) for histological analysis. The sections were stained with hematoxylin–eosin (H&E), the pathological changes of liver tissue were observed by fluorescence microscope, and LAS X software was used to analyze the image (Leica, Germany) (Heo and Song, 2011).

### Western blot analysis

About 50 mg of liver tissues was lysed with 10 times the volume of 1× RIPA lysis buffer (Beyotime Biotechnology, Shanghai, China). After centrifugation for 12,000 r/min, 5 min, the supernatant was collected and the protein concentration in the samples was determined by the BCA (Beyotime Biotechnology, Shanghai, China) method. The loading amount of each sample shall be approximately 50 μg according to the protein concentration. The protein sample was separated by SDS-PAGE gels and then transferred to a PVDF membrane, blocking the membrane with 5% nonfat milk, and the primary antibodies of p38-MAPK, p-p38MAPK (Cell Signaling Technology, Danvers, MA, USA) were used for Western blot. Then, the membrane was treated with an appropriate secondary antibody for 1 h at 37°C. Finally, the protein band information was visualized using a chemiluminescence system (Bio-Rad, USA), and Image analysis software (GeneTools from Syngene) was used to quantify the immune blots.

### Statistical analysis

Data were analyzed by using GraphPad Prism 5.0 and expressed as mean ± SD. The quantitative data were analyzed by one-way ANOVA. Differences were considered significant if *p* < 0.05.

## Results

### Determination of iridoid glycosides by HPLC

#### HPLC methodology validation

**FIGURE 2:**
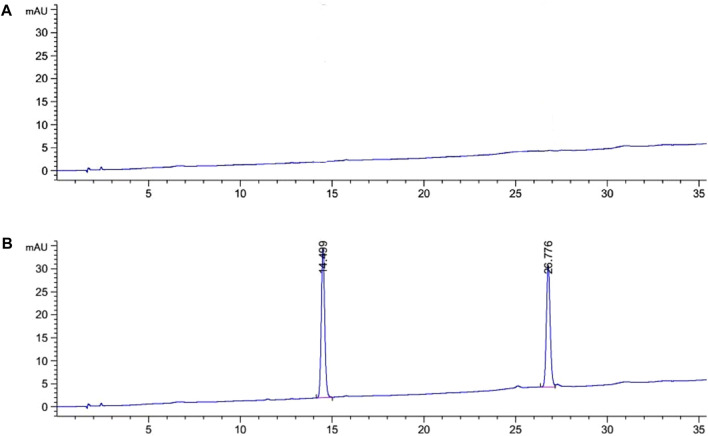
The HPLC chromatograms of mixed reference solution [**(A)** blank chromatograms; **(B)** the mixed reference chromatograms].

**System adaptability test:** The results of the system adaptability test are shown in Figure 2. It can be seen from the figure that the retention times of Specnuezhenide and Nuezhenoside G13 were 14.5 and 26.8 min, respectively. The peak separation degree was good, and the peak shape was normal.

**FIGURE 3:**
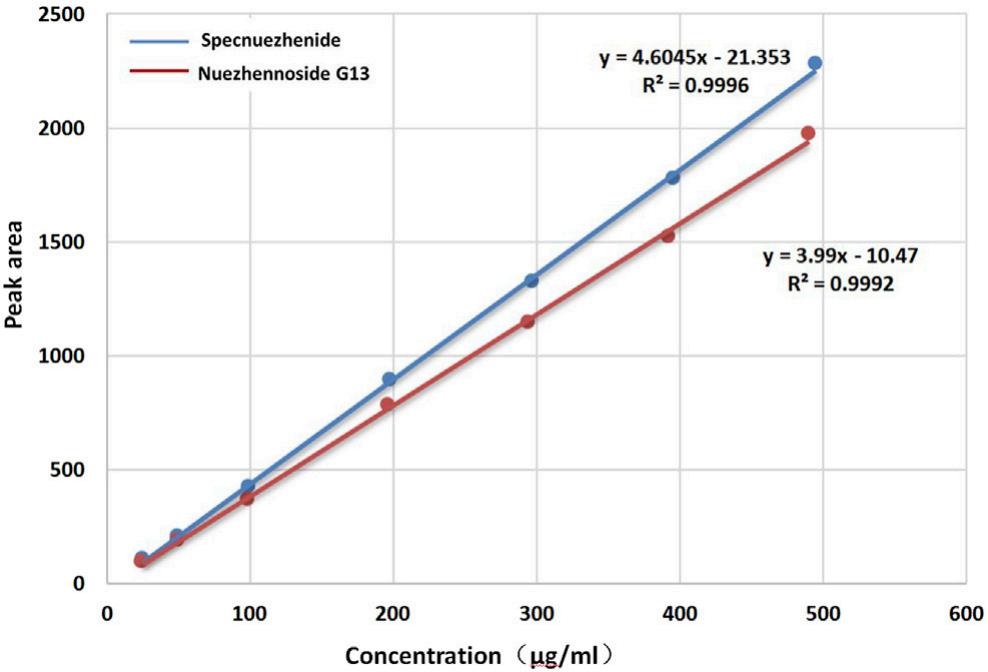
The standard curve of Specnuezhenide and Nuezhenoside G13.

**Linearity and range:** The results of references’ concentrations and peak areas are shown in Table 1. The standard curves were drawn with the concentration of the reference substance as the X-coordinate and the peak area as the Y-coordinate. The regression equation of Specnuezhenide was Y = 4.60X−21.35 (R^2^ = 0.9996), and the regression equation of Nuezhenoside G13 was Y = 3.99X−10.47 (R^2^ = 0.9992). The results showed that the linear relationship of Specnuezhenide was good in the range of 24.72–494.41 μg/ml and Nuezhenoside G13 in the range of 24.50–490.00 μg/ml (Figure 3).

**TABLE 1:** The linear test results of specnuezhenide and Nuezhenoside G13.

The test solution	Specnuezhenide			Nuezhenoside G13		
	Concentration (μg/ml)	Peak area	Average peak area	Concentration (μg/ml)	Peak area	Average peak area
1	24.72	106.7	108.1	24.50	95.5	98.3
		109.5			101.0	
2	49.44	207.3	207.3	49.00	194.9	189.2
		207.2			183.4	
3	98.88	426.1	425.1	98.00	374.7	369.8
		424.0			364.9	
4	197.76	896.0	893.8	196.00	775.6	782.3
		891.5			789.0	
5	296.65	1,322.5	1,326.7	294.00	1,150.2	1,148.4
		1,330.8			1,146.5	
6	395.53	1,772.1	1,778.6	392.00	1,521.9	1,521.8
		1,785.0			1,521.7	
7	494.41	2,286.5	2,282.0	490.00	1,975.4	1,975.5
		2,277.4			1,975.6	

**Precision test:** The precision test results of Specnuezhenide and Nuezhenoside G13 are shown in Table 2. The RSD values of the peak areas of Specnuezhenide and Nuezhenoside G13 were 0.53% and 1.78%, respectively. The results indicate that the precision was good.

**TABLE 2:** The precision test results of Specnuezhenide and Nuezhenoside G13.

Peak area	1	2	3	4	5	6	Average	RSD (%)
Specnuezhenide	424.7	422.0	426.1	427.6	428.7	427.9	426.2	0.53
Nuezhenoside G13	370.0	378.0	389.2	371.7	380.1	371.1	376.7	1.78

**FIGURE 4:**
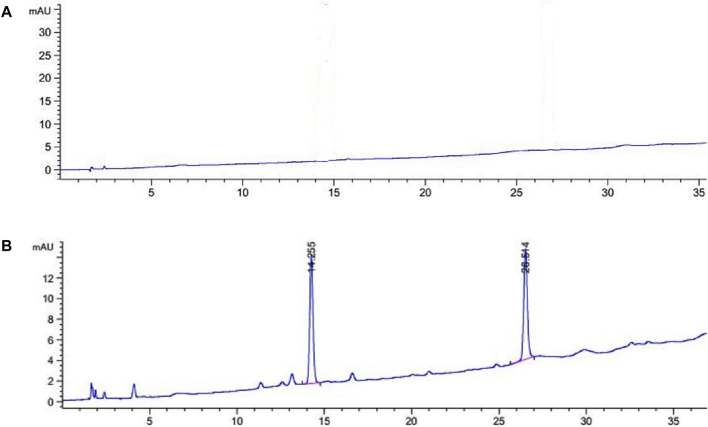
The HPLC chromatograms of OFSN [**(A)** blank chromatograms; **(B)** the OFSN chromatograms].

**Repeatability test:** The HPLC spectrum of OFSN is illustrated in Figure 4. The repeatability test results of Specnuezhenide and Nuezhenoside G13 are shown in Table 3. The RSD values of the contents of Specnuezhenide and Nuezhenoside G13 in OFSN were 0.75% and 1.36%, respectively, suggesting that the method had good repeatability.

**TABLE 3:** The repeatability test results of Specnuezhenide and Nuezhenoside G13.

Sample	Number	Concentration (mg/ml)	Peak area 1	Peak area 2	Average peak are	Content (%)	Average content (%)	RSD (%)
Specnuezhenide	1	1.000	159.8	158.4	159.1	39.20	38.99	0.75
	2	1.014	159.4	159.9	159.6	38.78		
	3	1.025	162.4	164.2	163.3	39.13		
	4	1.013	162.2	161.2	161.7	39.25		
	5	1.011	161.1	159.9	160.5	39.07		
	6	1.028	159.6	162.1	160.8	38.50		
Nuezhenoside G13	1	1.000	148.5	148.7	148.6	39.88	39.65	1.36
	2	1.014	151.6	148.2	149.9	39.65		
	3	1.025	149.0	151.6	150.3	39.32		
	4	1.013	152.6	151.9	152.2	40.27		
	5	1.011	151.9	150.0	151.0	40.02		
	6	1.028	145.2	151.8	148.5	38.77		

**Recovery test:** The recovery test results of Specnuezhenide and Nuezhenoside G13 are shown in Table 4 and Table 5, respectively. The recoveries of Specnuezhenide and Nuezhenoside G13 at low, medium, and high concentrations ranged from 95% to 105%, and the RSD values were less than 2%. It reflects that the method is feasible.

**TABLE 4:** The recovery test results of specnuezhenide.

Group	Sample content (mg)	Addition content (mg)	Measured content (mg)	Measured addition content (mg)	Recovery (%)	Average recovery (%)	RSD (%)
Low	0.3950	0.3164	0.7056	0.3106	98.17	99.35	1.66
	0.3950	0.3164	0.7071	0.3121	98.65		
	0.3950	0.3164	0.7153	0.3203	101.23		
Medium	0.3950	0.3955	0.7925	0.3975	100.50	100.59	0.26
	0.3950	0.3955	0.7920	0.3970	100.39		
	0.3950	0.3955	0.7940	0.3990	100.88		
High	0.3950	0.4746	0.8502	0.4552	95.92	95.34	1.22
	0.3950	0.4746	0.8511	0.4561	96.10		
	0.3950	0.4746	0.8411	0.4461	94.00		

**TABLE 5:** The recovery test results of Nuezhenoside G13.

Group	Sample content (mg)	Addition content (mg)	Measured content (mg)	Measured addition content (mg)	Recovery (%)	Average recovery (%)	RSD (%)
Low	0.4709	0.3724	0.8484	0.3775	101.36	101.76	0.98
	0.4709	0.3724	0.8471	0.3762	101.02		
	0.4709	0.3724	0.8541	0.3832	102.90		
Medium	0.4709	0.4704	0.9416	0.4707	100.06	99.18	1.84
	0.4709	0.4704	0.9431	0.4722	100.38		
	0.4709	0.4704	0.9276	0.4567	97.08		
High	0.4709	0.5684	1.0599	0.5890	103.62	103.21	0.58
	0.4709	0.5684	1.0591	0.5882	103.49		
	0.4709	0.5684	1.0536	0.5827	102.52		

**Stability test:** The stability test results of Specnuezhenide and Nuezhenoside G13 are shown in Table 6. The RSD values of the contents of Specnuezhenide and Nuezhenoside G13 in OFSN were 0.48% and 0.35%, respectively. It shows that the test solutions have good stability when placed at room temperature for 36 h.

**TABLE 6:** The stability test results of Specnuezhenide and Nuezhenoside G13.

Sample	Time (h)	Peak area 1	Peak area 2	Average peak area	Content (%)	Average content (%)	RSD (%)
Specnuezhenide	0	161.9	162.9	162.4	39.92	39.66	0.48
	8	160.4	160.7	160.6	39.51		
	16	160.2	160.9	160.6	39.51		
	32	161.1	161.5	161.3	39.68		
Nuezhenoside G13	0	153.4	148.1	150.8	40.41	40.40	0.35
	8	150.3	152.6	151.4	40.59		
	16	148.7	151.5	150.1	40.25		
	32	151.5	149.6	150.6	40.36		

The validation test results of HPLC methodology show that the method meets the requirements of system adaptability, linearity, precision, repeatability, recovery, and stability, implying that the method is suitable for determining the content of iridoid glycosides in the extraction of *Osmanthus fragrans*.

#### Determination of sample content

The percentage contents of Specnuezhenide and Nuezhenoside G13 in OFSE, OFSN, OFSN-3, and OFSN-4 are shown in Table 7. The content of iridoid glycosides in *Osmanthus fragrans* seeds’ ethanol extraction (OFSE) was relatively low. After extraction with n-butanol, the content of iridoid glycosides was significantly increased in *Osmanthus fragrans* seed’s n-butanol extraction (OFSN). After purification with D101 macroporous resin, the concentration of iridoid glycosides was further enriched. Specnuezhenide was mainly enriched in 30% ethanol elution portion (OFSN-3), and Nuezhenoside G13 was mainly enriched in 50% ethanol elution portion (OFSN-4).

**TABLE 7:** The percentage contents of Specnuezhenide and Nuezhenoside G13 in OFSE, OFSN, OFSN-3, and OFSN-4.

Sample	Specnuezhenide (%)	Nuezhenoside G13 (%)
OFSE	19.21	23.65
OFSN	39.20	39.88
OFSN-3	82.56	—
OFSN-4	12.04	87.94

### Liver and spleen indexes

Table 8 results suggested that the liver and spleen indexes of the model group were significantly increased compared with the control group (*p* < 0.01). Compared with the model group, the liver and spleen indexes of the positive control group were significantly decreased (*p* < 0.01); the liver index (*p* < 0.01) and spleen index (*p* < 0.05) were significantly decreased in the medium-dose group and the high-dose group.

**TABLE 8:** Effects of OFSN on liver and spleen index in mice (‾x ± s).

Group	*n*	Liver indexes (g/100 g)	Spleen indexes (g/100 g)
Control	10	4.69 ± 0.43	0.30 ± 0.04
Model	10	5.84 ± 0.40**	0.39 ± 0.08**
Prednisolone	10	4.87 ± 0.34^##^	0.30 ± 0.04^##^
OFSN-L	10	5.60 ± 0.48	0.37 ± 0.06
OFSN-M	10	5.15 ± 0.48^##^	0.32 ± 0.04^#^
OFSN-H	10	5.00 ± 0.38^##^	0.32 ± 0.06^#^

Data are mean ± SD; *p < 0.05 vs. control group; ^#^ *p* < 0.05 vs. model group.

### Biochemical detection

#### Effects of OFSN on SOD and MDA in liver homogenate of mice

As shown in Table 9, compared with the control group, the content of SOD was decreased significantly (*p* < 0.01) and the MDA was increased significantly (*p* < 0.01) in the model group. Compared with the model group, prednisolone and OFSN could increase the SOD and decrease the MDA to different degrees. Among them, the prednisolone and the high-dose OFSN could increase the SOD significantly (*p* < 0.05); the prednisolone and the middle- and high-dose OFSN could decrease the MDA significantly (*p* < 0.01).

**TABLE 9:** Effects of OFSN on biochemical detection of mice (‾x ± s).

Group	*n*	SOD (U/mgprot)	MDA (nmol/mgprot)	ALT (U/gprot)	AST (U/gprot)	TNF-α (pg/ml)	IFN-γ (pg/ml)
Control	10	285.04 ± 6.92	3.12 ± 0.37	49.06 ± 11.43	83.24 ± 14.28	272.04 ± 31.66	124.13 ± 19.34
Model	10	250.04 ± 19.52**	5.11 ± 0.55**	97.55 ± 20.28**	193.56 ± 37.95**	544.55 ± 68.26**	770.52 ± 63.15**
Prednisolone	10	279.14 ± 33.93^#^	3.63 ± 0.43^##^	57.69 ± 14.18^##^	100.38 ± 23.62^##^	374.21 ± 51.41^##^	209.30 ± 62.22^##^
OFSN-L	10	255.03 ± 26.19	4.51 ± 0.65	86.95 ± 19.83	180.94 ± 26.29	512.76 ± 34.24^#^	711.27 ± 80.21
OFSN-M	10	265.20 ± 29.62	4.11 ± 0.50^##^	75.59 ± 13.33^#^	163.61 ± 18.54^#^	444.59 ± 74.62^##^	576.24 ± 52.73^##^
OFSN-H	10	285.04 ± 6.92^#^	3.12 ± 0.37^##^	60.17 ± 10.30^##^	151.76 ± 20.12^##^	401.60 ± 46.27^##^	357.53 ± 47.44^##^

Data are mean ± SD; *p < 0.05 vs. control group; ^#^ *p* < 0.05 vs. model group.

#### Effects of OFSN on ALT, AST, TNF-α, and IFN-γ in plasma of mice

The effects of OFSN on ALT, AST, TNF-α, and IFN-γ in the plasma of mice are shown in Table 9. Compared with the control group, the levels of ALT and AST in the model group were significantly increased (*p* < 0.01). Compared with the model group, the levels of ALT and AST in the prednisolone group and the OFSN-L, OFSN-M, and OFSN-H groups were reduced to different degrees. Among them, statistically significant differences appeared in the prednisolone group (*p* < 0.01) and the OFSN-M (*p* < 0.05) and OFSN-H groups (*p* < 0.01).

Compared with the control group, the levels of TNF-α and IFN-γ in the model group were significantly increased (*p* < 0.01). Compared with the model group, prednisolone and different doses of OFSN could reduce TNF-α significantly (*p* < 0.01, *p* < 0.05); in addition, prednisolone and the middle- and high-dose OFSN could reduce IFN-γ significantly (*p* < 0.05).

### HE stains

**FIGURE 5:**
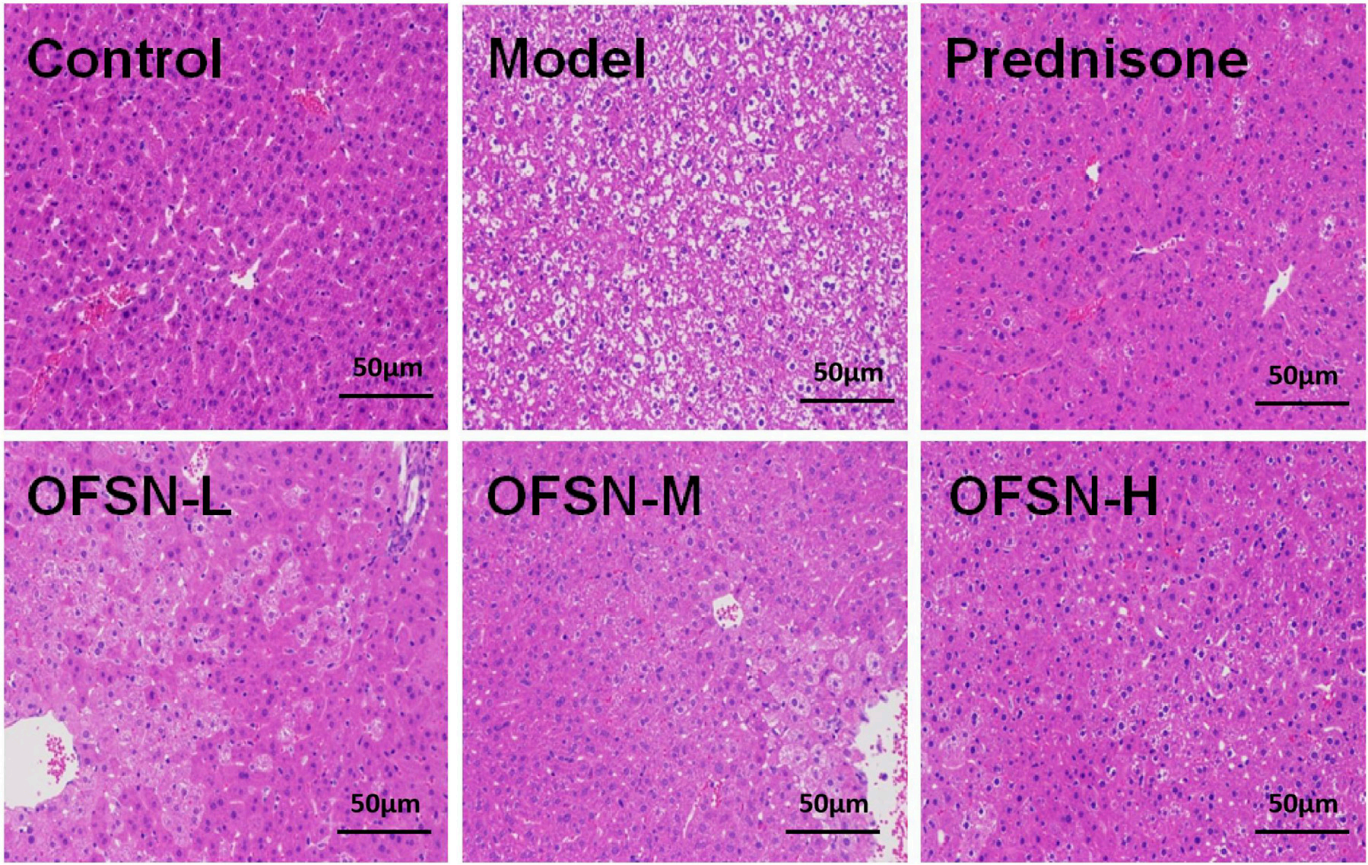
Pathological changes of liver tissues (HE staining, ×200).

The optical microscopy examination shows that no significant abnormalities were found in the liver tissue of the control group. The cytoplasm of hepatocytes was uniformly red, the nuclei were normal in size, and the nuclei were lightly stained. The hepatocytes around the terminal hepatic vein were radially arranged regularly, the hepatic sinuses were clear, and the cords were neatly arranged. The pathological examination of the model mice showed extensive hepatocyte swelling, loose cytoplasm and vacuolation, light cytoplasm staining, binuclear and polyploid nuclei, and liver plate disappearance. However, different doses of OFSN could reduce the pathological changes of the liver in mice to different degrees, especially those in the high-dose group (Figure 5).

### Western blot

**FIGURE 6:**
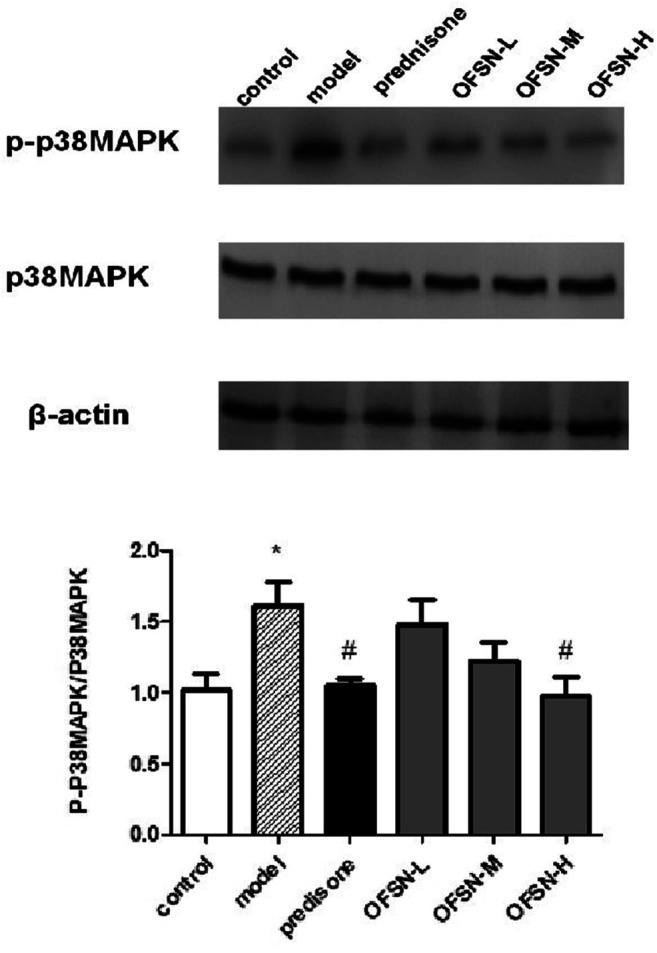
Effects of OFSN on p-p38MAPK/p38MAPK expression in liver tissues. Data are mean ± SD; *p < 0.05 vs. control group; ^#^ *p* < 0.05 vs. model group.

Compared with the control group, the expression level of p-p38MAPK in the model group significantly increased, with a statistically significant difference (*p* < 0.05). Compared with the model group, the expression levels of p-p38MAPK in the prednisone group and the OFSN group showed different decreasing degrees, among which the prednisone group and the OFSN-H group had statistical significance (*p* < 0.05) (Figure 6).

## Discussion

Liver is the biggest substantial viscera of the human body, playing an important role to the normal function of the body. However, as the modern environment changes and the pace of life accelerated, the liver becomes more and more vulnerable to damage. When liver parenchymal cells are damaged, the secondary changes of liver function will severely affect the normal performance of other organs and body functions. With the rapid development of modern medicine, more in-depth studies have been conducted on various aspects of liver disease. Meanwhile, people’s desire for safer and more effective drugs has become more urgent. Studies in recent years have shown that traditional Chinese medicine has suggested certain activities in the treatment of liver injury diseases, especially iridoid glycosides. Iridoid glycosides extracted from traditional Chinese medicine such as *Paederia scandens (Lour.) Merr. var. tomentosa (Rubiaceae)* (Peng et al., 2015), *Veronica ciliata Fisch* (Hua et al., 2020), gardenia (Chen et al., 2016), and rhubarb (Zhu et al., 2014) showed some protective effects on chemical and alcoholic liver injury. However, few studies on immune liver injury were found.

Immune liver injury is a chronic progressive inflammatory disease of the liver mediated by an autoimmune response (Kubes and Jenne, 2018). Its clinical manifestations present varying degrees of serum transaminase elevation, high γ-globulinemia, and positive autoantibodies, and in severe cases, cirrhosis and liver failure can develop rapidly. At present, the clinical treatment is glucocorticoid alone or in combination with azathioprine (Hardtke-Wolenski et al., 2012; Fan et al., 2019). However, long-term use of hormone drugs may produce severe adverse effects on the human body. Thus, it is important to find safer and more effective drugs. According to modern research reports, natural drugs have good efficacy in improving liver function and inhibiting the release of inflammatory mediators (Ding et al., 2018).

Most of the experimental studies on autoimmune liver injury were based on the mouse liver injury model induced by *Bacillus* Calmette–Guerin vaccine (BCG) combined with lipopolysaccharide (Yao and Yue, 2005), concanavalin A (Xu et al., 2006), D-galactosamine (Yang et al., 2016), etc. Among them, the process of liver injury induced by concanavalin A tail vein injection is similar to the pathological process of human acute immune liver injury (such as human acute viral hepatitis) and has dose dependence and organ specificity. Concanavalin A is a phytohemagglutinin that induces immune liver injury by increasing IFN-γ secretion following activation of T lymphocytes and by stimulating excessive release of TNF-α from macrophages (Trautwein et al., 1998). In addition, IFN-γ is a crucial activator of macrophages and plays a vital role in stimulating macrophages to secrete TNF-α and promoting Kupffer cells in the liver to participate in the inflammatory response. Studies revealed that early administration of TNF-α blockers could completely block the liver injury induced by D-galactosamine in mice (Lai et al., 2019). Therefore, TNF-α and IFN-γ are crucial for the development of immune liver injury induced by concanavalin A in mice (Fayad et al., 2005). The spleen is an important immune organ of the body, and its organ index can reflect the immune function of the body to a certain extent (Li et al., 2017). Immune liver injury caused by concanavalin A is organ-specific. When the body’s liver is damaged, inflammatory mediators will be released in excess, resulting in increased liver weight. Therefore, liver and spleen index measurement can be used to roughly estimate the success of the model establishment and the efficacy of the test products.

Besides, large amounts of oxygen free radicals are produced in liver cells during liver injury (Jaeschke, 2011). These oxygen free radicals act on the lipids of the cell membrane, triggering lipid peroxidation, producing MDA, and thus exacerbating membrane damage and causing ALT and AST, which are originally in the cytoplasm of liver cells and mitochondria, to enter the blood. Therefore, the level of ALT and AST in plasma is a good indicator to judge the severity of liver damage. By measuring the content of SOD and MDA in the liver homogenate, the strength of antioxidant resistance and the degree of damage to the membrane system can be understood (Di Meo and Venditti, 2020).

Mitogen-activated protein kinase (MAPK) cascade is one of the important signal transduction systems in cells and can be activated by various signals and distributed in the cytoplasm (Braicu et al., 2019). It has the dual phosphorylation ability of serine and tyrosine and participates in various biological reactions of cells after being activated by double phosphorylation. As an important signaling pathway of the MAPK family, p38MAPK can affect the production of a variety of cytokines and plays an important role in various physiological and pathological processes such as cell inflammation, proliferation, stress, apoptosis, cell cycle, and growth (Risco and Cuenda, 2012). Studies have shown that stimulation of macrophages with lipopolysaccharides (LPS) can activate intracellular p38MAPK to phosphorylate it (Wu et al., 2018), and the inhibition of this phosphorylation can reduce or even completely block the production of TNF-α in macrophages (Braicu et al., 2019), indicating that the production of TNF-α in the inflammatory response is closely related to the activation of p38MAPK.

Iridoid glycosides are a class of monoterpenoids, which widely exist in a variety of plants (Gousiadou et al., 2015). As a landmark component of Oleaceae plants, they also have a certain content in *Osmanthus fragrans* seeds. In this experiment, we extracted iridoid glycosides from *Osmanthus fragrans* seeds and determined the contents of Specnuezhenide and Nuezhenoside G13 by UV and HPLC methods. The results demonstrated that *Osmanthus fragrans* seeds are rich in Specnuezhenide and Nuezhenoside G13, and the linearity, precision, repeatability, recovery, and stability of HPLC method all met the requirements by validated with methodology.

Iridoid glycosides have a variety of biological activities such as anti-inflammatory and analgesic, hepatoprotective and cholagogic, bacteriostatic, sedative, antihypertensive, and antitumor. Therefore, on this basis, we studied the potential protective effect of *Osmanthus fragrans* seed extract on concanavalin A-induced immune liver injury in mice.

First, the mouse immune liver injury model was established by tail intravenous concanavalin A (20 mg/kg). Then, the contents of serum ALT, AST, IFN-γ, and TNF-α and the contents of liver tissue MDA and SOD were tested, the pathological changes of liver were observed by HE staining, and the expression levels of p38MAPK and p-p38mapk in liver tissue were detected by WB. The results of animal experiments showed that OFSN could significantly reduce the liver and spleen indexes, reduce the ALT and AST contents in plasma and the MDA content in liver tissue, and increase the SOD content. In addition, OFSN could also reduce the plasma IFN-γ and TNF-α levels. HE staining results suggested that the pathological changes in the liver tissues of mice treated with OFSN were alleviated to different degrees, while the WB results reflected that OFSN could significantly inhibit the expression of p-p38mapk.

The results showed that *Osmanthus fragrans* seeds are rich in iridoid glycosides; the HPLC method is stable and repeatable. OFSN has a good protective effect on mouse immune liver injury caused by concanavalin A. The mechanism may be related to inhibition of the phosphorylation of p38MAPK, inhibition of the release of inflammatory mediators, improvement of the antioxidant capacity of liver cells, and weakening of the occurrence of lipid peroxidation.

## Data Availability

The original contributions presented in the study are included in the article/Supplementary Material. Further inquiries can be directed to the corresponding authors.
